# Designing and evaluating a web-based self-management site for patients with type 2 diabetes - systematic website development and study protocol

**DOI:** 10.1186/1472-6947-12-57

**Published:** 2012-06-24

**Authors:** Catherine H Yu, Janet Parsons, Muhammad Mamdani, Gerald Lebovic, Baiju R Shah, Onil Bhattacharyya, Andreas Laupacis, Sharon E Straus

**Affiliations:** 1 Keenan Research Centre, Li Ka Shing Knowledge Institute, St. Michael's Hospital, 30 Bond Street, Toronto, ON M5B 1W8, Canada; 2 Department of Medicine, University of Toronto, Toronto, Canada; 3 Dhalla Lana School of Public Health, University of Toronto, Toronto, Canada; 4 Applied Health Research Centre, Li Ka Shing Knowledge Institute, St. Michael’s Hospital, 30 Bond Street, Toronto, ON M5B 1W8, Canada; 5 Department of Physical Therapy, University of Toronto, Toronto, Canada; 6 Department of Health Policy, Management and Evaluation, University of Toronto, Toronto, Canada; 7 Institute for Clinical Evaluative Sciences, G1 06, 2075 Bayview Avenue, Toronto, ON M4N 3M5, Canada; 8 Sunnybrook Research Institute, Sunnybrook Health Sciences Centre, Toronto, Canada; 9 Department of Family and Community Medicine, University of Toronto, Toronto, Canada

**Keywords:** Diabetes mellitus, Self care, Patient education, Self-efficacy, Medical informatics, Intervention development, Study protocol, User-Computer Interface, Repeated measures modeling, Qualitative methods

## Abstract

**Background:**

Given that patients provide the majority of their own diabetes care, patient self-management training has increasingly become recognized as an important strategy with which to improve quality of care. However, participation in self management programs is low. In addition, the efficacy of current behavioural interventions wanes over time, reducing the impact of self-management interventions on patient health. Web-based interventions have the potential to bridge the gaps in diabetes care and self-management.

**Methods:**

Our objective is to improve self-efficacy, quality of life, self-care, blood pressure, cholesterol and glycemic control and promote exercise in people with type 2 diabetes through the rigorous development and use of a web-based patient self-management intervention. This study consists of five phases: (1) intervention development; (2) feasibility testing; (3) usability testing; (4) intervention refinement; and (5) intervention evaluation using mixed methods. We will employ evidence-based strategies and tools, using a theoretical framework of self-efficacy, then elicit user feedback through focus groups and individual user testing sessions. Using iterative redesign the intervention will be refined. Once finalized, the impact of the website on patient self-efficacy, quality of life, self-care, HbA1c, LDL-cholesterol, blood pressure and weight will be assessed through a non-randomized observational cohort study using repeated measures modeling and individual interviews.

**Discussion:**

Increasing use of the World Wide Web by consumers for health information and ongoing revolutions in social media are strong indicators that users are primed to welcome a new era of technology in health care. However, their full potential is hindered by limited knowledge regarding their effectiveness, poor usability, and high attrition rates. Our development and research agenda aims to address these limitations by improving usability, identifying characteristics associated with website use and attrition, and developing strategies to sustain patient use in order to maximize clinical outcomes.

## Background

Management of diabetes is complex, and involves controlling the multiple risk factors that lead to complications. Given that patients provide the majority of their own diabetes care [1], patient self-management training is an important strategy with which to improve quality of care [1]. Several systematic reviews have examined the impact of self-management interventions on glycemic control, cardiac risk factors and psychological outcomes. These reviews have demonstrated positive effects on knowledge [2], self-reported dietary habits [2], quality of life [3], and glycemic control [2,4]. However, effects of interventions on lipids, physical activity, weight, and blood pressure were variable [2]. Characteristics of effective interventions include patient collaboration and regular reinforcement [2].

Despite this sound rationale and evidence supporting these interventions, participation in self management programs is low [5]. In addition, the efficacy of behavioural interventions wanes over time [6], reducing the impact of self-management interventions on patient health. Consumers are increasingly accessing the World Wide Web and social media as sources of health information [7,8]. Web-based interventions have the potential to improve diabetes care and self-management. Two systematic reviews suggest that web-based media improve knowledge and understanding [9,10], social support [10], behaviour change [10] and clinical outcomes [10] for a variety of diseases. However effective education and self-management principles have not been systematically incorporated into existing diabetes websites for patients to optimize these interventions. A review of existing diabetes websites demonstrated that these websites were characterized by didactic information of variable quality, high reading levels, and a newspaper-format display, with little interactive technology, social support or problem-solving assistance [11,12]. A recent systematic review of electronic diabetes-related tools found that they had moderate but inconsistent effects on a variety of psychological and clinical outcomes including HbA1c and weight and a high prevalence of usability errors [13]. It also found that more interactive tools resulted in continued website use and greater clinical improvement.

Thus, we sought to improve effective self-management, as evidenced by improved self-efficacy, quality of life, self-care, blood pressure, cholesterol and glycemic control and exercise promotion, in people with type 2 diabetes through the use of a web-based patient self-management intervention that addresses the identified limitations of existing web-based interventions. Specifically, we sought to ensure inclusion of evidence-based content in a usable format, with incorporation of interactivity, social support and problem-solving assistance, patient collaboration and regular reinforcement.

## Methods

Intervention development requires careful planning and the use of theory-based strategies to increase the probability of effectiveness, programme adoption and implementation [14]. Graham et al’s Knowledge to Action framework [15] was chosen for evaluating this knowledge translation activity (Table 1) because it is context-focused, enables knowledge-producer and knowledge-user collaboration, and emphasizes sustainability. The Knowledge to Action framework incorporates the need to adapt the knowledge to fit with the local context, which is particularly important for this activity: this project is occurring in the context of the provincial government’s 5-year diabetes initiative, which includes a web-based dissemination strategy. Secondly, this framework is particularly useful for emphasizing the collaboration between knowledge producers and knowledge users throughout the process and for facilitating the use of research knowledge by several stakeholders, such as practitioners, policymakers, patients and the public. By involving stakeholders in the choice of question and interventions, the external validity of the research is strengthened. Finally, it addresses the need to sustain knowledge use by anticipating changes and adapting accordingly. As described above, sustainable knowledge use is essential given the chronic nature of diabetes. In this paper, we describe our innovative systematic approach to intervention development and evaluation.

**Table 1:** Knowledge-to-Action framework

**Knowledge to Action framework**[15]	**Study phase**
Identify problem. Identify, review, select knowledge	Patient self-management training has increasingly become recognized as an important strategy with which to narrow the care gap. Several systematic reviews have examined the impact of diverse self-management interventions and have demonstrated positive effects on knowledge self-reported dietary habits [6], quality of life [3] and glycemic control [4,6]. Despite the strength of this evidence base, participation in these programs is low. Given the growing prevalence of diabetes worldwide as well as the strong evidence base upon which available guidelines were developed, effectively bridging the knowledge to practice gap in this area has the potential to significantly improve health care outcomes and thus health care delivery and system sustainability.
Adapt knowledge to local context	In July 2008 the Ontario MOHLTC launched the Ontario Diabetes Strategy, to improve prevention and care for Ontarians with chronic diseases, starting with diabetes, through a mix of prevention, access to technology, personal planning and access to specialized resources and health professionals. All Ontarians with diabetes and their health care providers will be supported through a series of inter-related initiatives. As part of this larger initiative, there is an implementation plan for patient self-management tools, as well as a plan for measuring and reporting on improvements in clinical care and outcomes on a web-based patient portal.
Assess barriers to knowledge use	Barriers to knowledge use can occur at several levels, including the health care system, the health care team and organization, the health care profession, the patient and, finally, the guidelines or their education delivery system. Brown described a similar framework, and categorized barriers to diabetes care on three levels: organization, provider, and patient. Barriers at the patient level include acceptance of the diagnosis, education, self-motivation and adaptation to daily living. Poor adherence to guidelines may be a result of patient preferences, expectations or knowledge. Our initiative will focus on barriers at the patient level.
Select, tailor, implement interventions	(a) Selecting the intervention (Phase 1) Web-based interventions have the potential to bridge the gaps in diabetes care and self-management. Two systematic reviews suggest that web-based media improve knowledge or understanding [9,10], social support [10], behaviour change [10] and clinical outcomes [10] in a variety of disease states. (b) Tailoring the intervention. The intervention will be refined following feasibility (Phase 2) and usability testing (Phase 3) with patients with diabetes. (c) Implementing the intervention. The refined self-management tool (Phase 4) will be implemented in a pilot study (Phase 5).
Monitor knowledge use	A mixed method study, as described in the main text, will be conducted, consisting of an interrupted time series and individual interviews (Phase 5).
Evaluate outcomes	
Sustain knowledge use	Barriers to sustained knowledge use will be addressed in the planning phase of tool development, implementation and dissemination, and further explored upon completion of the pilot study with qualitative methodology (Phase 5).

### Study overview

This study consists of five phases: (1) intervention development; (2) feasibility testing; (3) usability testing; (4) intervention refinement; and (5) intervention evaluation using mixed methods (repeated measure modeling, individual interviews).

### Phase 1: Intervention development

Our objective was to create an evidence-based, theory-driven self-contained website. Based on our systematic review of electronic tools on topics relevant to diabetes [13], we selected tools that were known to be effective, relevant and usable. For example, we included multi-media diabetes education modules targeting individuals with low literacy that were demonstrated to be effective in lowering A1c [16] and that were easy to use [13]. In addition, we incorporated behavioral intervention strategies that were found to be effective in a systematic review of internet-based interventions, including stress management and communication tools [17]. For example, we included text- and graphic-based material on coping with diabetes, an online forum to communicate with peers, as well as tips and website-generated reports to facilitate communication with health care providers.

**Figure 1:**
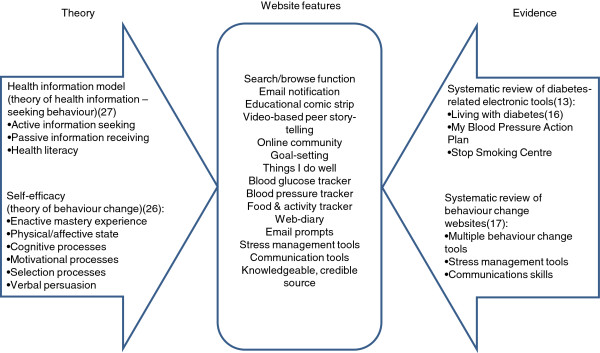
**Evidence and theory-based framework for intervention development.** Schematic depicting theoretical underpinnings (health information model, self-efficacy) and evidence base (systematic reviews of electronic tools, behavior change websites) contributing to website features.

We integrated these interventions with the theoretical framework of self-efficacy, a theory that has not only been validated in predicting and promoting patient behavior change but has also been demonstrated to improve clinical outcomes [18-25]. For example, diabetes self-management education programs incorporating self-efficacy have been shown in randomized controlled trials to improve knowledge [25], health behaviour [24,25], self-efficacy [23-25], HbA1c [23-25], and weight [25] and microvascular complications [24]. Briefly, self-efficacy refers to “beliefs in one’s capabilities to organize and execute the courses of action required to produce given attainments” [26]. It arises from one’s successes or failures during previous performances, observations of others’ experiences, verbal persuasion and physiological and affective states, and is mediated by cognitive, motivational, affective and selective processes. We used these sources and mediators of self-efficacy in formatting our site and selecting tools. For example, we incorporated feedback, goal-setting, peer story-telling, and monitoring tools into the website by including computer-generated responses to user entries, a goal-setting application, videos of peer testimonials, trackers for blood glucose, blood pressure, weight and physical activity and a diary) (Figure 1).

We also drew from the literature insights regarding patient health information seeking behavior to promote optimal use of the website [27]. Developed through qualitative focus group methodology, the approach of Longo et al. acknowledges the role of passive receipt and active retrieval of information, as well as the role of relationships with other individuals and health care providers in helping to process and interpret information. We used this model of health information seeking behavior to inform the development of interfaces that would enable a flexible approach to information seeking. To this end, we will automate email briefs of selected content, optimize search algorithms to enable self-directed retrieval, create an online forum to permit sharing of experiences, and develop tools (e.g. blood pressure trackers) and content (e.g. “How to prepare for your appointment with your physician”) to facilitate communication with health care providers (Figure 1).

We chose to create a self-contained website that did not require regular one-on-one input by a health care provider to optimize sustainability.

### Phase 2: Feasibility testing

We will employ focus group methodology to assess acceptability, usability, sustainability, strengths and weaknesses of this intervention. The website will be presented to participants who will be asked to work through the web based tools to complete a task that simulates real clinical use. The task invites participants to use the website to determine their personal risk of heart disease and specific strategies to reduce this risk, including the role of blood pressure in vascular risk and methods to reduce their blood pressure.

#### Participants

A purposive sampling strategy will be used to generate a heterogeneous group that captures the perspectives of patient participants with varied experiences. Participants (age greater than 25 years) with varied socio-demographic profiles (e.g. gender, age, use of insulin, ethnicity, educational attainment, annual income) and technological proficiency will be recruited from diabetes care centers at two academic health science centers in Toronto, Canada, and will complete a baseline questionnaire of user demographics. A sample size of 3 focus groups is estimated to achieve saturation of themes and to generate sufficient feedback to refine the tool; while sample size of 5–8 people is generally required for a homogeneous group, we wanted to sample a heterogeneous group as outlined above [28].

#### Outcomes

We will probe about intervention content, such as website title, general website organization, website searching tools, format of interactive and didactic tools, and content that they would find valuable. We are also interested in intervention processes, such as barriers and facilitators to the adoption of the specific tools and of the website generally, factors affecting continued use, reasons to visit the site repeatedly, the role of social networking, and comfort with entering personal information online. We want to determine features of the components that are perceived by participants to be valuable, and those which are in need of further development.

#### Data collection

Focus group sessions will employ semi-structured interview guides developed by team members with knowledge translation and qualitative research expertise [29]. Face and content validity of this guide will be assessed with team members and refined as needed. All focus group interviews will be digitally audio-recorded and field notes kept.

#### Data analysis

Audiotapes will be transcribed verbatim [30]. In keeping with qualitative methodology, data analysis will occur in conjunction with data collection [31]. Transcripts will be coded for emergent categories and themes using constant comparison [31]. All transcripts will be coded and reviewed independently by three team members with expertise in qualitative research methods; consensus on coding will be reached through comparison, discussion, and agreement among these three reviewers. Further, memos kept by the qualitative team members will be used to help monitor the emergent analytic framework and regular meetings will enable continued dialogue and discussion within the project [32]. NVivo software (version 9) will be used to assist data management and monitor the emerging analysis.

### Phase 3: Usability testing

We will conduct usability testing sessions using cognitive task analysis [33], whereby users are individually asked to “think aloud” as they perform specific tasks representing scenarios designed to cover the major functionalities of the web-based intervention in a one-hour session. We plan to do additional cycles of usability testing after intervention refinement in an iterative process of evaluation and redesign.

#### Participants

A consecutive sample of ten participants with type 2 diabetes aged greater than 25 years will be recruited from diabetes care centers at two academic health science centers in Toronto, Canada. The sample size should be sufficient, as research has shown that up to 80% of usability issues can be identified with 5 to 8 participants [33].

#### Outcomes

Data regarding paths users take to accomplish tasks, errors made, when and where they encountered confusion or frustration, time spent, and degree of satisfaction will be generated.

#### Data collection

A consultant with expertise in health informatics and human factors engineering will conduct each session, and further prompt users with a structured interview guide on usability. All usability testing sessions will be video and audio-taped. In addition, field notes will be kept of all sessions as a further source of data for analysis. Video tapes of the computer screen will be used to capture user actions in response to prompted tasks.

#### Data analysis

Audio tapes will be transcribed verbatim. Transcripts will be coded for emergent categories and themes using constant comparison [31]. Transcripts will be coded and reviewed independently by three team members with expertise in qualitative research methods, and consensus on the coding framework will be reached [34,35]. NVivo software (version 9) will be used to assist with data management and retrieval for analysis. Video tapes will be used in conjunction with audio tapes to identify the area of the website in discussion and user actions recorded.

### Phase 4: Intervention refinement

Based on data from the feasibility testing, refinements will be made to the website via ongoing discussion with the research and development team. Similarly, following usability testing, further refinements will be made to the website in an iterative process of testing and redesign. We anticipate that refinement will occur over a one-month period.

### Phase 5: Intervention evaluation

We hypothesize that a web-based patient self-management intervention, developed and revised based on our usability and feasibility testing, will result in improvements in self-care score, self-efficacy score, health care user satisfaction score and quality of life score in patients with type 2 diabetes with access to the internet.

#### Observational cohort study

##### Participants

A consecutive sample of individuals aged > 25 years with hemoglobin A1c (HbA1c) > 7.0%, systolic blood pressure (sBP) > 130 mmHg, low-density-lipoprotein cholesterol (LDL-C) > 2.0 mmol/L or a body mass index (BMI) > 25 kg/m^2^ will be recruited from 2 family practice units and 2 endocrinology clinics at 2 urban academic health science centers in Toronto. Those with active heart disease (defined as Canadian Cardiovascular Society class 3 to 4 angina), who were non-English-speaking, who were not available for follow-up, or who had no regular access to the telephone or internet will be excluded. Age, gender, ethnicity, education, employment, duration of diabetes, complications, smoking status, anti-hyperglycemic, antihypertensive, and lipid-lowering agents, HbA1c, sBP, LDL-C, weight, current use and comfort with computer and internet, self-care score, self-efficacy score, health care user satisfaction score and quality of life score will be obtained at baseline.

#### Outcomes

##### Primary outcome

The primary outcome, self efficacy, will be assessed with the Modified Grossman Self-efficacy for Diabetes Scale which has moderate to high reliability (Cronbach’s alpha = 0.51 to 0.86) [36,37] (Table 2).

**Table 2:** Description of primary outcome scales

**Outcome**	**Scale**	**Description**
Self-efficacy	Modified Grossman Self-efficacy for Diabetes Scale [36,37]	The scale contains 25 items that measure the intensity of self-efficacy for activities of the diabetes regimen. Subjects are asked to describe how much they believe they could or could not do what was stated. The responses on this 6-point scale range from “*very sure I can’t*” to “*very sure I can*” do what was stated in each item. Higher scores indicate greater confidence in one’s ability to perform the designated treatment activities. The following statements are examples of the self-efficacy items: “Figure meals and snacks at home” and “Keep track of blood sugar levels”. The modified self-efficacy scale has a moderate to high reliability (Cronbach’s alpha = 0.51 to 0.86).
Self-care behavior	Summary of Diabetes Self-Care Activities Measure – Revised [38,39]	Items selected from this self-report instrument assess participants’ frequency (over the past 7 days) of engaging in diabetes self-care behaviors, including following a healthy diet, spacing out carbohydrates evenly across the day, physical activity, self-monitoring of blood glucose testing, foot care, and medication and/or insulin taking. For each diabetes self-care behavior, participants are asked to respond using the following prompt: “*On how many of the last 7 days*…” Responses, which are based on a 7-day week, range from 0 days to 7 days. Greater number of days indicated better self-management. Reliability and validity for this instrument have been found to be adequate, with a test-retest correlation of 0.40 and internal consistency of 0.47.
Diabetes-specific quality of life	Diabetes Distress Scale [40]	The DDS is a 17 item instrument that assesses emotional distress and functioning specific to living with diabetes. Responses are scored on a 6-point Likert-type scale from 1 = “*no problem”* to 6 = “*serious problem”*. Scores can range from 17–102 with higher scores indicating poorer diabetes-related quality of life and lower scores indicating better diabetes-related quality of life. The DDS has been found to have high internal reliability with a Cronbach’s alpha of 0.93, good convergent validity with the Center for Epidemiological Studies Depression Scale (CESD) (r = 0.56) and self-care behaviours including lower adherence to eating recommendations (r = 0.30, p < 0.001) and lower levels of physical activity (r = 0.13, p < 0.01) [40]. In addition, diabetes distress has been demonstrated to be associated with HbA1c (r = 0.17-0.31, *P =* .00-.001), diet (r = −0.38, *P =* .00), physical activity (r = −0.13, *P =* .01) and medication adherence (r = −0.16, *P =* .00) [41,42].

##### Secondary outcomes

Secondary outcomes include self care and quality of life, which will be assessed through well-validated scales, described below and in Table 2.

*Self-care behavior* will be assessed with the Summary of Diabetes Self-Care Activities Measure – Revised. Reliability and validity for this instrument have been reported to be fair, with a test-retest correlation of 0.40 and internal consistency of 0.47 [38,39].

*Diabetes-specific quality of life* will be assessed with the Diabetes Distress Scale which has been found to have high internal reliability with a Cronbach’s alpha of 0.93, good convergent validity with the Center for Epidemiological Studies Depression Scale (CESD) (r = 0.56) and self-care behaviors including lower adherence to eating recommendations (r = 0.30, p < 0.001) and lower levels of physical activity (r = 0.13, p < 0.01) [40]. In addition, diabetes distress has been demonstrated to be associated with HbA1c (r = 0.17-0.31, *P =* .00-.001), diet (r = −0.38, *P =* .00), physical activity (r = −0.13, *P =* .01) and medication adherence (r = −0.16, *P =* .00) [41,42].

These patient-based outcomes were selected because they are direct and relevant measures of knowledge use by patients. Diabetes self-care is a direct measure of knowledge use, and self-efficacy was chosen in order to better understand the mediating variables of knowledge use. Quality of life was chosen as a more holistic and patient-centered measure of impact of knowledge use.

HbA1c, systolic and diastolic blood pressure, LDL-C and weight to be collected every 3 months; these outcomes were chosen to help inform sample size calculations of future prospective trials.

##### Data collection

Data on outcomes will be collected by patient-completed questionnaires; patients will be compensated with a small honorarium for each questionnaire. For the pre-implementation phase, aggregates of patient data will be obtained every three weeks for nine months, resulting in 12 data points. The intervention will then be implemented. For the post-implementation phase, aggregates of patient data will be obtained every three weeks for nine months following the intervention, resulting in another 12 data points. HbA1c and LDL-C will be collected from the hospital medical records. Systolic and diastolic blood pressure will be collected by the research coordinator with an Omron Blood Pressure Model (Model no. HEM-907XL) and recorded as the average of 3 readings. Weight will be collected by the research coordinator with a Seca digital scale (Model 707). Web server log analysis will be done to assess frequency and duration of the specific components of the intervention; metrics selected are described in Appendix C. All participants will be asked at the end of the study to disclose whether other web-based interventions were used, and if so, whether they employed text- or image-based didactic materials, interactive technology or behavioral strategies.

##### Sample size calculation

Using a range of correlations from 0.2-0.8, a 0.05 significance level, and a power of 80%, a sample of at most 52 subjects is required to detect a change of 0.5 units in self-efficacy score before the intervention as compared to after. A formula for paired mean comparisons was used [43] and the longitudinal nature of the study will only increase the power [44]. The sample size was further adjusted to account for a 40% expected dropout rate (attrition).

##### Data analysis

Descriptive analysis will examine the distribution of each variable of interest and determine which variables are suitable as covariates for modeling. In addition, exploratory and graphical measures will be used for each of the primary and secondary outcomes to determine the distribution of the outcome of interest to enable a suitable statistical model to be constructed. Should the distribution of the outcome not be ideal, a suitable transformation will be used to enable proper statistical modeling [45,46]. The primary analysis, assessing the effect of the intervention on self-efficacy, will be examined using a linear mixed model to account for inherent correlation of the repeated measures within each individual [47]. The model will examine the effect of intervention on the outcome, self-efficacy, while adjusting for the following variables: age, ethnicity, education level, employment status, income (above or below $30 000) and health literacy. Should the data support it the model will also include interactions of these variables with the intervention to determine whether the impact of the intervention on self-efficacy varies for different levels of the variables. Time will also be included in the model to adjust for any trends over time. Model diagnostics will be performed using residual analysis.

Similar modeling will be used for the other outcomes of self-care and quality of life as well as A1C, systolic BP, diastolic BP, LDL and weight. For secondary outcomes, due to the data being collected less frequently, fewer variables will be included in the model. Each model in the secondary analysis will examine the effect of the intervention while adjusting for self-efficacy, income and ethnicity. In addition to these covariates, the outcomes systolic BP, diastolic BP and LDL will also include age as a covariate while the analysis examining weight as the dependent variable will also include insulin use as a covariate. Should the data support it these variables will also be examined as interaction terms with the intervention.

This analysis approach will allow us to examine the effect of the intervention on each of the primary and secondary outcomes while adjusting for other important variables and accounting for the correlation that arises in repeated measures data.

Other variables of interest such as website usage statistics, website user satisfaction and subscales of self-efficacy, self-care and quality of life will be examined using descriptive and graphical analysis.

#### In-depth interviews

Following completion of the repeated measures study, in-depth focused, individual interviews will be used to assess acceptability, usability, strengths and weaknesses of the intervention, facilitators and barriers to its use, user satisfaction, and sustainability of use.

##### Participants

A purposive sample of participants (based on gender, age, ethnicity, duration of diabetes, educational attainment, annual income, pre-intervention experience with website use) from the repeated measures study will be recruited by the study coordinator. A sample size of 25 participants is likely to be necessary to achieve theoretical saturation.

##### Outcomes

The perceived impact of each web tool on participants’ knowledge of diabetes self-management, perceptions regarding their experiences of diabetes and how it impacts their lives, stage of behavior change, perceived self-efficacy and self-management will be assessed, prompted by open-ended questions from the interviewer. Participants’ perspectives regarding barriers and facilitators to the adoption and sustained use of a web-based intervention, types of information included and formats used (and preferred) will be discussed. Accounts of their use of other health information resources, their interaction with the healthcare system, and their perceptions of online privacy and sharing will also be explored.

##### Data collection and analysis

All interviews will be audio tapes and transcribed verbatim [30]. Transcripts will be coded for emergent categories and themes using constant comparison [31]. Transcripts will be coded and reviewed independently by three team members with expertise in qualitative research methods. A coding framework will be developed based on the emerging analysis, and then tested and refined with subsequent interviews in an iterative process. The coding framework will be based on consensus amongst the analysts [34,35]. NVivo software (version 9) will be used to assist with data management and retrieval for analysis. In addition, the qualitative data will be compared with the quantitative data in order to triangulate and interpret both datasets in the context of the whole study [48].

### Research ethics

The study was approved by the Research Ethics Board of St. Michael’s Hospital (reference number 09–091) and Sunnybrook Health Sciences Centre (reference number 177–2009).

## Discussion

### Principal results

While the first two phases of this study will shed light on information needs of patients with type 2 diabetes, our subsequent phases will assess the impact of this systematically developed intervention. Specifically, the repeated measures design will determine the impact of web-based self-management programs on important psychological and clinical outcomes. We will also identify factors (both of participants and of the website) that correlate with website use and effectiveness in order to guide future intervention development and research.

### Strengths and limitations

Strengths of our approach include our rigorous theory-driven and evidence-based approach to intervention development, our systematic refinement of the intervention based on feasibility and usability data, our use of mixed methods for evaluation, and our selection of usability assessment techniques*.* The importance of theory was highlighted in a recent systematic review which found that effectiveness of internet-based interventions is associated with more extensive use of theory [17]. Similarly, evidence-based content should form the foundation of an educational website, yet pre-existing diabetes websites have wide variations in the quality of evidence leveraged [11]. Finally, stakeholder input and usability testing are key to intervention implementation and sustainability [49] but are frequently neglected, with up to 60% of diabetes-related websites having at least three usability errors [13]. These elements of design – theory, evidence, stakeholder input, and usability testing - are fundamental to intervention effectiveness and should represent the gold standard for intervention development [13].

Within the qualitative evaluation, we will use trained moderators who are not otherwise invested in the project, collect background information regarding participants to ensure that there is diverse representation, and use a sound methodology for coding and interpreting the data into relevant themes, including independent coding by two individuals and interpretation by three individuals to ensure data trustworthiness [50]. In addition, triangulation of the qualitative findings with the quantitative results will promote methodological rigor [48]. One limitation of our usability testing approach is that this technique may miss consistency problems (for example, conventions, such as an arrow indicating “next”, not being used consistently throughout the website). However, we will address this issue by ensuring that our sessions are moderated and analyzed by a usability expert.

Potential limitations of our evaluation study include the consistency with which participants will respond at each time point, generalizability, as well as maturation and history threats to internal validity. Response rate will be maximized with the use of reminder emails and honoraria. We will maximize generalizability by recruiting broadly across all socio-demographic strata and by collecting data to allow us to characterize our study populations. We will account for maturation threats by assessing for interaction with time, and we will assess for history threats by recording secular events that could impact on our outcomes prospectively throughout the study period and by asking our participants on their exit questionnaires.

## Conclusions

Web-based interventions to improve self-management of diabetes show great promise because they target patient behaviors directly, can be easily scaled up given the fixed cost of website development and are inexpensive to maintain. This study builds on previous work using the most rigorous approach to intervention development, including usability testing, qualitative studies of acceptability, iterative refinement, and measures the impact on self-efficacy, quality of life and risk factor control using a repeated measures design. Our intervention is based robustly on a theoretical foundation of self-efficacy, which has been demonstrated to predict and promote behavior change and improve health outcomes in chronic diseases such as diabetes mellitus. In addition, use of behavior change theory itself in intervention development is associated with greater intervention effectiveness. This, in combination with evidence-based content and strategies, stakeholder input and usability testing, should result in an optimally engineered website designed to improve psychological and clinical outcomes and complement health care delivery. Increasing use of the World Wide Web by consumers for health information and ongoing revolutions in social media are strong indicators that consumers are primed to welcome a new era of technology in health care. However, their full potential is hindered by limited knowledge regarding their effectiveness, high prevalence of usability errors, and high attrition rates. Our development and research agenda aims to address these limitations by assessing effectiveness, addressing usability errors, identifying characteristics associated with website use and attrition, and developing strategies to reduce website attrition in order to maximize clinical outcomes.

## Abbreviations

HbA1c: Hemoglobin A1c; sBP: Systolic blood pressure; LDL-C: Low-density-lipoprotein cholesterol; BMI: Body mass index.

## Competing interests

The authors declare that they have no competing interests.

## Authors’ contributions

CY conceived of the study and drafted the manuscript. All authors participated in the design of the study, revised the manuscript critically for intellectual content, and have read and approved the final manuscript.

## Pre-publication history

The pre-publication history for this paper can be accessed here:

http://www.biomedcentral.com/1472-6947/12/57/prepub
